# Anterior insular cortex mediates hyperalgesia induced by chronic pancreatitis in rats

**DOI:** 10.1186/s13041-019-0497-5

**Published:** 2019-09-04

**Authors:** Yang Bai, Li-Tian Ma, Yan-Bing Chen, Dan Ren, Ying-Biao Chen, Ying-Qi Li, Hong-Ke Sun, Xin-Tong Qiu, Ting Zhang, Ming-Ming Zhang, Xi-Nan Yi, Tao Chen, Hui Li, Bo-Yuan Fan, Yun-Qing Li

**Affiliations:** 1 Department of Anatomy, Histology and Embryology & K. K. Leung Brain Research Centre, Fourth Military Medical University, No. 169, West Chang-le Road, Xi’an, 710032 China; 2 Department of Gastroenterology, Tangdu Hospital, Fourth Military Medical University, Xi’an, 710032 China; 3 Department of Anatomy, Fujian Medical University, Fuzhou, 350108 China; 4 Department of Anatomy, Guangxi Medical University, Nanning, 530021 China; 5 Department of Anatomy, Fujian Health College, Fuzhou, 350101 China; 6 Department of Cardiology, The Second Affiliated Hospital of Xian Jiaotong University, Xian Jiaotong University, Xi’an, 710004 China; 7 Joint Laboratory of Neuroscience at Hainan Medical University and Fourth Military Medical University, Hainan Medical University, Haikou, 571199 China

**Keywords:** Rat, Chronic pancreatitis, Anterior insular cortex, Long-term potentiation, Excitatory synaptic transmission, Hyperalgesia, Anxiety

## Abstract

Central sensitization plays a pivotal role in the maintenance of chronic pain induced by chronic pancreatitis (CP), but cortical modulation of painful CP remains elusive. This study was designed to examine the role of anterior insular cortex (aIC) in the pathogenesis of hyperalgesia in a rat model of CP. CP was induced by intraductal administration of trinitrobenzene sulfonic acid (TNBS). Abdomen hyperalgesia and anxiety were assessed by *von* Frey filament and open field tests, respectively. Two weeks after surgery, the activation of aIC was indicated by FOS immunohistochemical staining and electrophysiological recordings. Expressions of VGluT1, NMDAR subunit NR2B and AMPAR subunit GluR1 were analyzed by immunoblottings. The regulatory roles of aIC in hyperalgesia and pain-related anxiety were detected via pharmacological approach and chemogenetics in CP rats. Our results showed that TNBS treatment resulted in long-term hyperalgesia and anxiety-like behavior in rats. CP rats exhibited increased FOS expression and potentiated excitatory synaptic transmission within aIC. CP rats also showed up-regulated expression of VGluT1, and increased membrane trafficking and phosphorylation of NR2B and GluR1 within aIC. Blocking excitatory synaptic transmission significantly attenuated abdomen mechanical hyperalgesia. Specifically inhibiting the excitability of insular pyramidal cells reduced both abdomen hyperalgesia and pain-related anxiety. In conclusion, our findings emphasize a key role for aIC in hyperalgesia and anxiety of painful CP, providing a novel insight into cortical modulation of painful CP and shedding light on aIC as a potential target for neuromodulation interventions in the treatment of CP.

## Introduction

Sustained abdominal pain is the most prominent feature of chronic pancreatitis (CP). It takes a heavy toll on the well-being of CP patients and renders a tough challenge for both gastroenterologists and pain physicians [1]. The management dilemma reflects a paucity of our knowledge concerning the pathogenesis of painful CP. The past decade has witnessed a conceptual transition from anatomical basis to neurobiological theory [2]. A growing body of evidence concerning the peripheral or spinal mechanisms of pancreatic pain identified a host of molecular targets, such as transient receptor potential vanilloid1 (TRPV1) and nerve growth factor (NGF), but corresponding therapeutic alternatives have not been applied into clinical practice owing to the lack of clinical evidence [3]. More importantly, owing to the highly-plastic nature of cortical synapses, enhanced peripheral inputs from the inflammatory pancreas trigger aberrant central pain processing, which perpetuates painful CP even after surgery or drugs aiming at eliminating peripheral barrage [4, 5]. Considering these, we should embrace the ideology of cortical processing during painful CP.

Cerebral cortex is the ultimate hub for pain perception and sensitization, mediating the discriminative, affective and cognitive dimensions of pain [6]. Insights from electroencephalogram [7–9] and imaging studies [10, 11] provide evidence of structural and functional reorganizations of cerebral cortex, especially bilateral insular cortex (IC) and anterior cingulate cortex (ACC), in CP patients. IC is critical for the sensation, regulation and chronification of pain. Long-term potentiation (LTP) of insular excitatory synaptic transmission is believed to mediate pathological pain [12, 13]. Glutamate is the major excitatory neurotransmitter within IC. Basic synaptic transmission is mainly mediated by α-amino-3-hydroxy-5-methyl-4-isoxazole propionic acid receptor (AMPAR), while insular LTP requires both *N*-methyl-D-aspartate receptor (NMDAR) and AMPAR, which play important roles in the induction and expression phases, respectively [13]. Excitatory synaptic transmission within IC was enhanced during neuropathic pain, which was attributable to enhanced presynaptic release of glutamate and postsynaptic recruitment of AMPAR and NMDAR [14, 15]. Thus, both insular lesion [16, 17] and drugs aiming at inhibiting insular glutamatergic transmission [14, 15, 18, 19] or plastic changes [20] exerted analgesic effects in neuropathic and inflammatory pain models. Recently, emerging evidence indicated the role of IC in stress-related visceral hypersensitivity [21–23]. However, whether there exist such neuroplastic changes within IC under the condition of painful CP receives few attentions.

Patients afflicted by CP usually exhibit pain-related comorbidities, such as emotional disorders and locomotor dysfunction [24, 25]. Unfortunately, much less focus has been directed on the relief of the affective dimension of pancreatitis pain in both clinical and preclinical researches. As a critical hub for both somatosensory and viscerosensory processing, IC is also important for emotional feelings, including emotional pain modulation [26, 27]. It is commonly recognized that anterior IC (aIC) is more involved in the emotional dimension of pain while posterior IC (pIC) participates in the sensory aspect of pain [28, 29]. Considering these, we propose that aIC plays an important role in visceral hypersensitivity and pain-related negative emotions in painful CP, which may provide important clues for clinical rehabilitative strategies for painful CP.

In the present work, we adopted abdominal withdraw threshold (AWT) and exploratory behavior in the open field as an indicator of anxiety, to evaluate the sensory and emotional aspects of painful CP, respectively. Electrophysiological approaches were performed to explore CP-induced insular neuroplastic changes. Immunoblottings were applied to unravel the molecular basis for these plastic changes. Behavioral tests together with pharmacologic and chemogenetic methods aiming at manipulating the activity of aIC were performed to demonstrate its role in the pathogenesis of painful CP.

## Materials and methods

### Animals and experimental design

A total of 137 male *Sprague-Dawley* rats (250–280 g) were used, which were provided by the Experimental Animal Center of the Fourth Military Medical University (Xi’an, China). All protocols were performed according to the guidelines of the International Association for the Study of Pain [30] and approved by the Institutional Animal Care and Use Committee of the Fourth Military Medical University. Animals were provided water and food *ad libitum* 12 h before and after induction of pancreatitis, during which food and water were withdrawn.

Experiment 1: Evidence of pancreatitis-induced abdomen hyperalgesia: 27 rats were equally divided into 3 groups (naïve, sham and TNBS groups). Pancreatitis was induced by intraductal infusion of 2% trinitrobenzene sulfonic acid (TNBS; Sigma, St. Louis, MO, USA) as previously reported [31]. Sham rats received intraductal infusion of equal volume of saline. Naïve group received no surgery. AWT, as a measure of referred abdominal mechanical hypersensitivity, was an indirect marker of visceral sensitization [31]. In this experiment, AWT was monitored by *von* Frey filaments (VFF; Stoelting, Kiel, WI, USA) assay at post-operation day (POD) 3, 7, 14, 21, 28 and 35, respectively.

Experiment 2: Evidence of pancreatitis-induced hypolocomotion and effect of painful CP on the expressions of insular FOS, VGluT1, NR2B and GluR1. 53 rats were divided into 8 groups (6–7 rats in each group), which were exposed to intraductal TNBS or saline treatment. Open field test was performed to evaluate the locomotion and anxiety behavior according to our previous work [32] at POD 7, 14 and 28, respectively. After behavioral tests, rats were sacrificed. Heart blood was sampled and then centrifuged at 6000 g for 10 min. Serum specimens were measured by amylase, lipase and total bilirubin activity assay kits (Sigma). Meanwhile, at least 3 rats in each group were processed for aIC sampling for VGluT1, NR2B and GluR1 immunoblotting. Other rats were perfused with 4% paraformaldehyde (PFA). Brain tissues were then sampled for FOS immunostaining and pancreatic tissues were sampled for H&E staining.

Experiment 3: Effect of painful CP on insular excitatory synaptic transmission. 16 rats were equally divided into 2 groups (sham and TNBS groups), which were exposed to intraductal saline or TNBS treatment, respectively. At POD 14, rats were sacrificed, and brain sections containing aIC were performed for electrophysiological recordings.

Experiment 4: Effect of CNQX and AP-5 microinjection into aIC on abdomen hypersensitivity of CP rats. 16 rats were divided into 3 groups (5 in saline group, 5 in CNQX group and 6 in AP-5 group), all of which were exposed to intraductal TNBS treatment. For drug microinjection and behavioral test, the experimental paradigm was described in Fig. 8a.

Experiment 5: Effects of inactivation of aIC neural activity on abdomen hypersensitivity and anxiety of CP rats. To non-specifically modulate the activity of aIC neurons, 13 rats were divided into 2 groups, and received chemogenetic virus injection within aIC (7 rats in hSyn-mCitrine group and 6 in hSyn-Gi group). To further explore the role of aIC pyramidal neurons in painful CP, 12 rats were equally divided into 2 groups (CaMKIIa-mCherry and CaMKIIa-Gi groups). All rats were exposed to intraductal TNBS treatment. For chemogenetic behavioral tests, the experimental designs were shown in Figs. 9a and 10a.

### Behavioral tests

For VFF testing, the belly area designated for stimulation was shaved 3 days before testing. Animals were habituated in the testing apparatus until calming down. VFFs with increased forces from 0.16 g to 26 g were applied to the abdomen 5 times, each for 5–8 s with 5 min interval. Minimal force eliciting at least 3 times withdrawn responses was considered as AWT.

For open field test, rats were placed in the center of the open field (100 cm × 100 cm × 60 cm) under dim lighting. A motion-tracking system (Shanghai Mobile Datum Information Technology, CO. Ltd., Shanghai, China) was utilized to record the movement trace for 15 min. Anxiety-like behavior was evaluated by total traveling distance and the traveling distance in the center of the open field.

### Pancreatic H&E staining

For the verification of pancreatitis, pancreatic tissues were immersed in 4% PFA overnight, then 30% sucrose solution overnight, subsequently transferred to progressive xylene washes and embedded in paraffin. Paraffin blocks were cut at 8 μm thickness and then stained with hematoxylin and eosin (H&E) to evaluate pathological changes.

### Immunohistochemical staining

On POD 14, TNBS and saline-treated rats were deeply anesthetized by i.p. administration of 7% chloral hydrate and then perfused with 50 ml of 0.01 M phosphate buffer saline (PBS, pH 7.4), followed by 500 ml 4% PFA fixative solution in 0.1 M phosphate buffer (PB, pH 7.4). After perfusion, brains were removed, placed in 30% sucrose solution for 24 h at 4 °C and then cut into coronal sections at 35 μm thickness into 6 dishes as 6 sets of every sixth serial sections. The sections containing aIC in the first and second sets were performed immunohistochemical staining for FOS and double immunofluorescent staining for VGluT1 and NeuN, respectively. The sections were incubated in 10% normal donkey serum (NDS) for 40 min at room temperature (RT) to block non-specific immunoreactivity, and then incubated overnight at 4 °C with following antibodies (shown in Table 1) in sequence: [1] primary antibodies for 18–24 h at 4 °C; [2] secondary antibodies for 4 h at RT; or [3] avidin-biotin complex for 2 h (for FOS staining) at RT. For FOS staining, sections were then reacted with 0.05 M Tris-HCl buffer containing 0.02% DAB (Dojin, Kumamoto, Japan) and 0.003% H_2_O_2_ for visualizing FOS. Finally, the slices were mounted onto glass slides, coverslipped for examination by light microscopy (AH-3, Olympus, Tokyo, Japan) for FOS staining or confocal microscope (CLSM, FV1000, Olympus) for double immunostaining. The images were captured and analyzed using Fluoview 1000 (Olympus). For FOS counting, 15 sections from anterior (Bregma + 3.00 ~ + 2.16), middle (Bregma + 1.22 ~ + 0.38) and posterior (Bregma − 1.14 ~ − 1.98) parts of IC (5 sections from each part) in one dish from each rat were counted for the total number of FOS-immunoreactive neurons in the three parts of IC between sham and TNBS groups (*n* = 3 rats in each group). For the determination of immunofluorescence intensity, nine microscopic images of aIC (with a 60 x objective lens) were taken from three sections per animal. The mean intensity of VGluT1 immunofluorescence of the nine images per rat was measured in Image J (*n* = 3 rats in each group).

**Table 1:** Antibodies used immunohistochemical staining

Antigens	Primary antibodies	Secondary antibodies	Tertiary antibodies
FOS	Mouse anti-FOS (1:500; Abcam, Cambridge, MA, USA)	Biotinylated donkey anti-mouse (1:500; Millipore, Billerica, MA, USA)	Avidin-biotin complex (1:200; ABC kit, Vector, Burlingame, CA, USA)
VGluT1/NeuN	Mouse anti-NeuN (1:200; Millipore, Billerica, MA, USA) Rabbit anti-VGluT1 (1:300; Sysy, Gottingen, NI, GER)	Alexa594-donkey anti-mouse (1:500; Invitrogen, Carlsbad, CA, USA) Alexa488-donkey anti-rabbit (1:500; Invitrogen, Carlsbad, CA, USA)	

### Western blotting

Insular tissues from four groups (sham, TNBS POD 7, 14 and 28 groups) were harvested in cold artificial cerebrospinal fluid (ACSF). Total protein (for VGluT1, NR2B and GluR1 assays) was prepared according to our previous study [33]. Membrane and cytoplasmic proteins (for NR2B, pNR2B, GluR1 and pGluR1 assays) were separated by Minute™ Plasma Membrane Protein Isolation Kit (Invent Biotechnologies, EdenPrairie, MN, USA) [34]. Subsequently, 30 μg protein from each sample (quantitatively measured by bicinchoninic acid protein assay; Thermo Scientific; Rockford, IL, USA) was subjected to 10% sodium dodecyl sulfate-polyacrylamide gel (SDS-PAGE) electrophoresis and electrophoretically transferred to polyvinylidene difluoride (PVDF) membranes (Immobilon-P, Millipore, Billerica, MA, USA). After blocking in 5% PBS-containing DifcoTM skim milk for 2 h, the membranes were incubated overnight at 4 °C with following primary antibodies: mouse anti-VGluT1 (1:500; Millipore); rabbit anti-pNR2B-Tyr^1472^(1:500; Millipore); rabbit anti-NR2B (1:500; Abcam, Cambridge, UK); rabbit anti-GluR1 (1:500; Millipore); rabbit anti-pGluR1-Ser^845^ (1:500; Millipore). The immunoblots were then incubated with corresponding horseradish peroxidase (HRP)-conjugated secondary antibodies (goat anti-rabbit or goat anti-mouse, 1:5000; Amersham Pharmacia Biotech, Piscataway, NJ, USA). To verify equal loading, we also probed the membranes with rabbit anti-GAPDH (1:5000; Beijing TDY BIOTECH CO., Beijing, China) and rabbit anti-N-cadherin (1:2000; Millipore) for cytosol and membrane extracts, respectively. Bands were visualized by enhanced chemiluminescence (ECL) detection method (Amersham Pharmacia Biotech) and exposed to film. The scanned images were quantified and analyzed with Image J software. Target protein levels were normalized against GAPDH or N-cadherin levels and expressed as fold changes relative to those of the sham group (*n* = 3 rats in each group).

### Extracellular field EPSP recordings

For brain slice preparations, rats were anesthetized with 7% chloral hydrate and coronal brain sections (300 μm) containing aIC were cut at 4 °C with a vibratome in oxygenated ACSF (in mM: 124 NaCl, 2.5 KCl, 2 MgSO_4_, 2 CaCl_2_, 25 NaHCO_3_, 1 NaH_2_PO_4_, 37 glucose, pH 7.4). Then, the sections were transferred to a recovery chamber with oxygenated ACSF at RT for 1 h for subsequent electrophysiological experiments.

For extracellular field excitatory postsynaptic potential (fEPSP) recordings, a commercial 64-channel recording system (MED64, Panasonic, Osaka, Japan) was used. The MED64 probe (P515A, Panasonic) has an array of 64 planar microelectrodes arranged in an 8 × 8 pattern (50 × 50 μm in size, interelectrode distance 150 μm), which could cover the insula (Fig. 3a, b). The preparation of MED64 probe was similar to our previous study [35]. The section was perfused with oxygenated ACSF at the rate of 2–3 ml/min with the aid of peristaltic pump (Minipuls®3, Inc., Gilson, France) during recording. After a 60-min recovery, one channel in the layers IV-V of aIC, from which the best synaptic responses could be elicited in surrounding recording channels, was selected as the stimulation site. Biphasic constant current pulses (8–24 mA, 0.1 ms) generated by the data acquisition software (Mobius, Panasonic Alpha-Med Sciences, Washington DC, USA) were applied to the stimulation site every minute. Testing intensity was determined when a half-maximal fEPSP was elicited in channels closest to the stimulation site. The fEPSPs evoked at both the superficial and deep layers were amplified by a 64-channel amplifier, displayed on the monitor screen, and stored on the hard disk of a microcomputer. After baseline synaptic responses were stabilized for 30 min, a theta burst stimulation (TBS) protocol (5 bursts at 5 Hz, repeated 5 times at 10 s intervals, 4 pulses at 100 Hz for each burst) was given at the test intensity to elicit post-synaptic LTP (post-LTP). After TBS, the test stimulus was repeatedly delivered every minute for 2 h to monitor the time course of LTP. For quantification of the LTP data, the fEPSPs measured by the slope (the rising phase between 10 and 90% of the peak response) and the amplitude were normalized as percent change from the baseline level. For comparison of LTP magnitude between two groups, the average value of the last 10 min recordings was compared statistically.

### Whole-cell patch-clamp recording

For whole-cell patch-clamp recordings, the section was placed in a recording chamber maintained with oxygenated ACSF at 28 °C and pyramidal neurons in the layers II-III of aIC were recorded. The potassium-based intracellular solution within the micropipettes (8–10 MΩ) contained the following (in mM): 120 K-gluconate, 5 NaCl, 0.2 EGTA, 10 HEPES, 2 MgATP, 0.1 Na_3_GTP, 1 MgCl_2_ and 10 phosphocreatine (adjusted to pH 7.2 with KOH, 290 mOsm). Spontaneous EPSCs (sEPSC) were recorded with membrane clamped at − 60 mV. To elicit synaptic responses in neurons within superficial layers, a bipolar tungsten stimulating electrode was placed within layer V (Fig. 4a). AMPA receptor-mediated EPSCs were elicited by repetitive stimulations every 10 s with neurons clamped at − 70 mV in the presence of 50 μM AP-5 (Tocris, Bristol, UK), while NMDA receptor-mediated responses were measured at 40 mV in the presence of 20 μM CNQX (Tocris). For paired-pulse facilitation (PPF) testing, the intervals were 35, 50, 75, 100 and 150 ms. To investigate the relationship between the spike number and the intensity of injected currents, pyramidal neurons were current-clamped and injected 50–350 pA depolarizing currents with a step-size of 50 pA. The rheobase current (minimum current to elicit an action potential) was explored via injecting depolarizing currents with a step-size of 2 pA.

For calculating the rectification of glutamate receptor-mediated EPSCs, cesium-based intracellular solution containing the following (in mM): 122 Cs-Gluconate, 5 TEA-Cl, 3.7 NaCl, 0.2 EGTA, 20 HEPES, 0.3 MgATP, 0.3 Na_3_GTP, 10 BAPTA, 0.1 spermine and 5 QX-314 (adjusted to pH 7.2 with CsOH, 290 mOsm) was used. The AMPA or NMDA receptor-mediated EPSCs were recorded at holding potentials of 50, 30, 10, 0, − 20, − 40 and − 60 mV. Then, the ratio of peak EPSC amplitudes at negative (− 40 mV) and positive (50 mV) holding potentials was measured as rectification index.

All experiments were conducted in the presence of picrotoxin (100 μM, Sigma) to block GABA_A_ receptor-mediated inhibitory synaptic currents. All recordings were performed with a MultiClamp 700B amplifier (Axon Instruments, Sunnyvale, CA, USA) and digitized at 5 kHz by DigiData 1550B (Axon Instruments). The access resistance of 10–30 MΩ was monitored throughout the experiment. Data were discarded if the access resistance changed by 15% during experiment.

### Drug microinjection and behavioral tests

7 days before TNBS treatment, rats were anesthetized and secured on a stereotaxic frame. Guide cannulas (26 gauge) were implanted bilaterally into aIC (AP: + 2.16, ML: ±5.0, DV: − 6.2 mm). On POD 14, intra-aIC injections were delivered through an injector cannula (30 gauge) which was located 0.2 mm lower than the guide. A Hamilton syringe (10 μl) was connected to the injector by a thin polyethylene tube and was driven by a motorized pump (ALCBIO, Shanghai, China). AP-5 (50 mM, 0.4 μl per side) or CNQX (20 mM, 0.4 μl per side) was infused into bilateral aIC at the rate of 0.05 μl/min, with an equivalent volume of saline as control. All injections were followed by an additional 5 min before removal of the injection cannula. AWT was measured before surgery, 30 min before and after drug microinjection at POD 14, as well as at POD 15. Injection sites were verified *post hoc* and rats with inaccurate sites were excluded.

### Chemogenetics and behavioral tests

To non-specifically inhibit the activity of aIC neurons, we injected hSyn promoter-driven recombinant AAV virus (rAAV2/9-hSyn-hM4Di-mCitrine, 0.4 μl/30 min, Taitool Bioscience, Shanghai, China) into bilateral aIC (AP: + 2.16, ML: ±5.0, DV: − 7.2 mm) 7 days before TNBS treatment. An equivalent amount of rAAV2/9-hSyn-mCitrine was injected into rats in sham group. To specifically inhibit the activity of aIC pyramidal neurons, we injected Calmodulin-dependent Protein Kinase IIa (CaMKIIa) promoter-driven virus (rAAV2/9-CaMKIIa-hM4Di-mCherry, 0.4 μl/30 min, BrainVTA, Hubei, China) into bilateral aIC 7 days before TNBS treatment. An equivalent amount of rAAV2/9-CaMKIIa-mCherry was injected into rats in sham group. On POD 14, clozapine-N-oxide (CNO, 3 mg/kg) was administrated intraperitoneally into the rats. AWT tests were performed before TNBS treatment, 1 h before and after CNO administration at POD 14, as well as at POD 15. Open field test was performed 2 h after CNO administration at POD 14. Injection sites were verified *post hoc* and rats with inaccurate sites were excluded.

### Data analysis

Results were expressed as means ± SEM. Statistical analyses between two groups were tested by Student’s *t*-test (SPSS 17.0). Statistical comparisons between multiple groups were made using one-way ANOVA or one-way repeated ANOVA followed by LSD *post-hoc* test. Analyzed numbers for each experiment are indicated in corresponding figures *P* < 0.05 was considered statistically significant.

## Results

### TNBS-induced abdomen hypersensitivity and anxiety

**Fig. 1:**
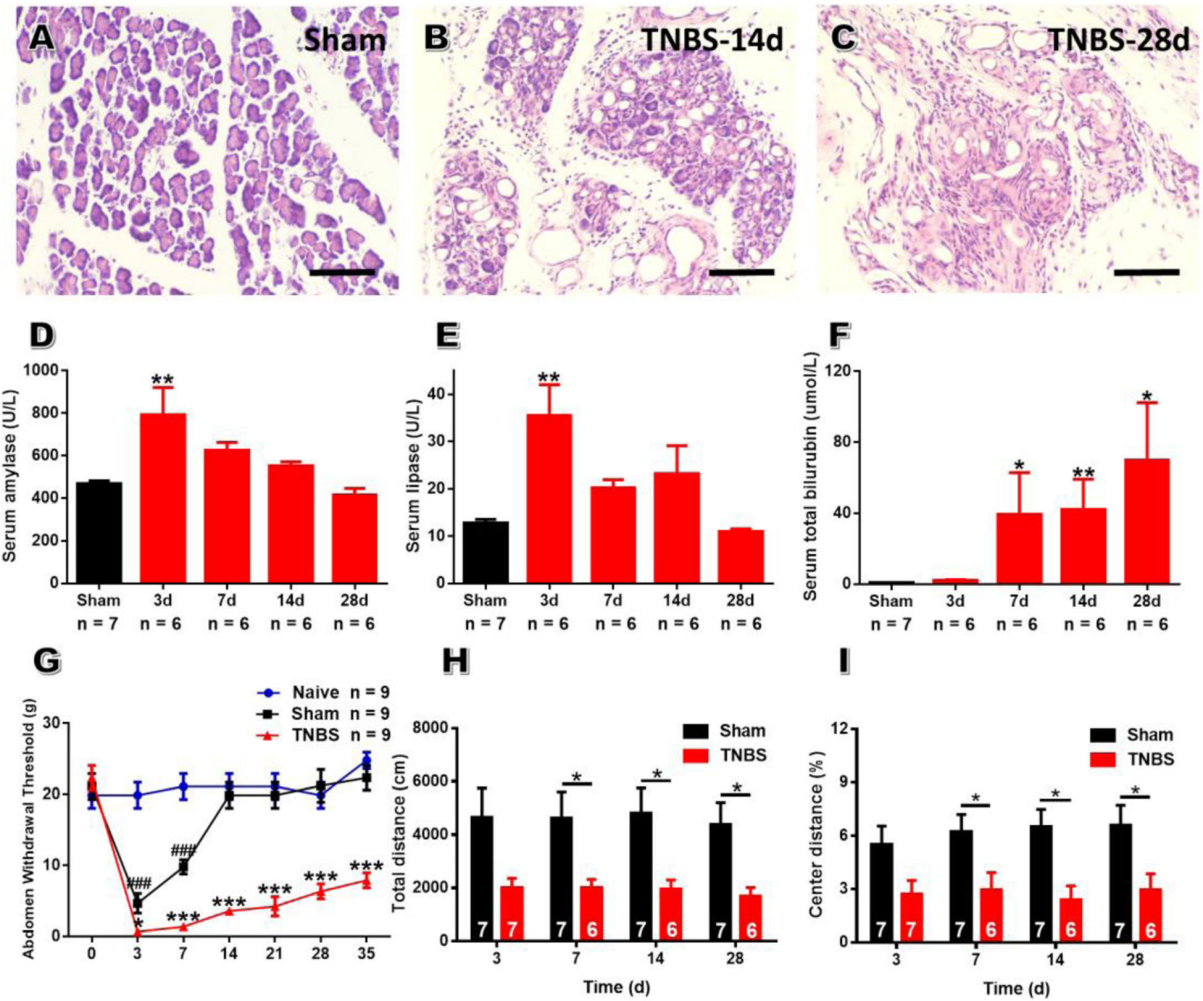
TNBS-treated rats exhibit abdomen hyperalgesia and anxiety-like behaviors. **a-c**: Representative pancreas histology of sham group (**a**), TNBS-treated group on POD 14 (**b**) and POD 28 (C). Bar = 100 μm. **d-f**: TNBS treatment increased serum content of amylase (**d**), lipase (**e**) and total bilirubin (**f**) compared to sham rats along the course of CP, one-way ANOVA in (**d**, **e**) and Kruskal-Wallis test in (**f**). **g**: TNBS-treated rats showed decreased abdomen withdrawal threshold along the course of CP, while rats with sham surgery exhibited transient abdomen mechanical hypersensitivity which returned to baseline on POD 14, one-way repeated ANOVA. **h-i**: Rats with CP traveled less distance in the open field (h) as well as in the center of the open field (**i**) than sham rats from POD 7 to 28, unpaired *t*-test. Numbers within the bars denote the numbers of rats in each group, * *P* < 0.05, ** *P* < 0.01, *** *P* < 0.001, TNBS vs sham; ^###^ *P* < 0.001, sham vs naïve

The validity of CP model was evidenced by histopathological changes of pancreatic tissue, such as acinar atrophy, inflammatory infiltration and stromal fibrosis (Fig. 1a-c). Owing to the damage of acinar cells, TNBS-treated rats exhibited increased contents of serum amylase and lipase on POD 3, which decreased along the course of CP and returned to baseline on POD 28 (Fig. 1d, e). In addition, TNBS-treated rats also exhibited high level of serum total bilirubin from POD 7 to 28 (Fig. 1f). All these changes mimicked those appeared in human chronic pancreatitis [36].

Then, behavioral assays were performed to provide evidence of abdomen hypersensitivity in CP rats. Sham group exhibited transient decrease of AWT which returned to baseline on POD 14, possibly owing to the abdominal incision during surgery. Comparing to sham group, TNBS-treated rats displayed a prolonged decrease in AWT from POD 7 to 35 (Fig. 1g). Open field testing showed that CP rats traveled less distance in the open field from POD 7 to 28, implicating the existence of hypolocomotion (Fig. 1h). Further analyses of open field results showed that CP rats traveled less distance in the central area along the course of CP, suggesting the generation of anxiety-like emotions in CP rats (Fig. 1i). All these performance in the open field were consistent with those of mice with irritable bowel syndrome (IBS) observed in our previous study [32]. Considering these, apart from AWT as an indirect indicator of visceral hypersensitivity, we adopted hypolocomotion and decreased exploratory behavior to measure emotional pain modulation of IC in CP rats.

### TNBS treatment increases FOS expression within IC

**Fig. 2:**
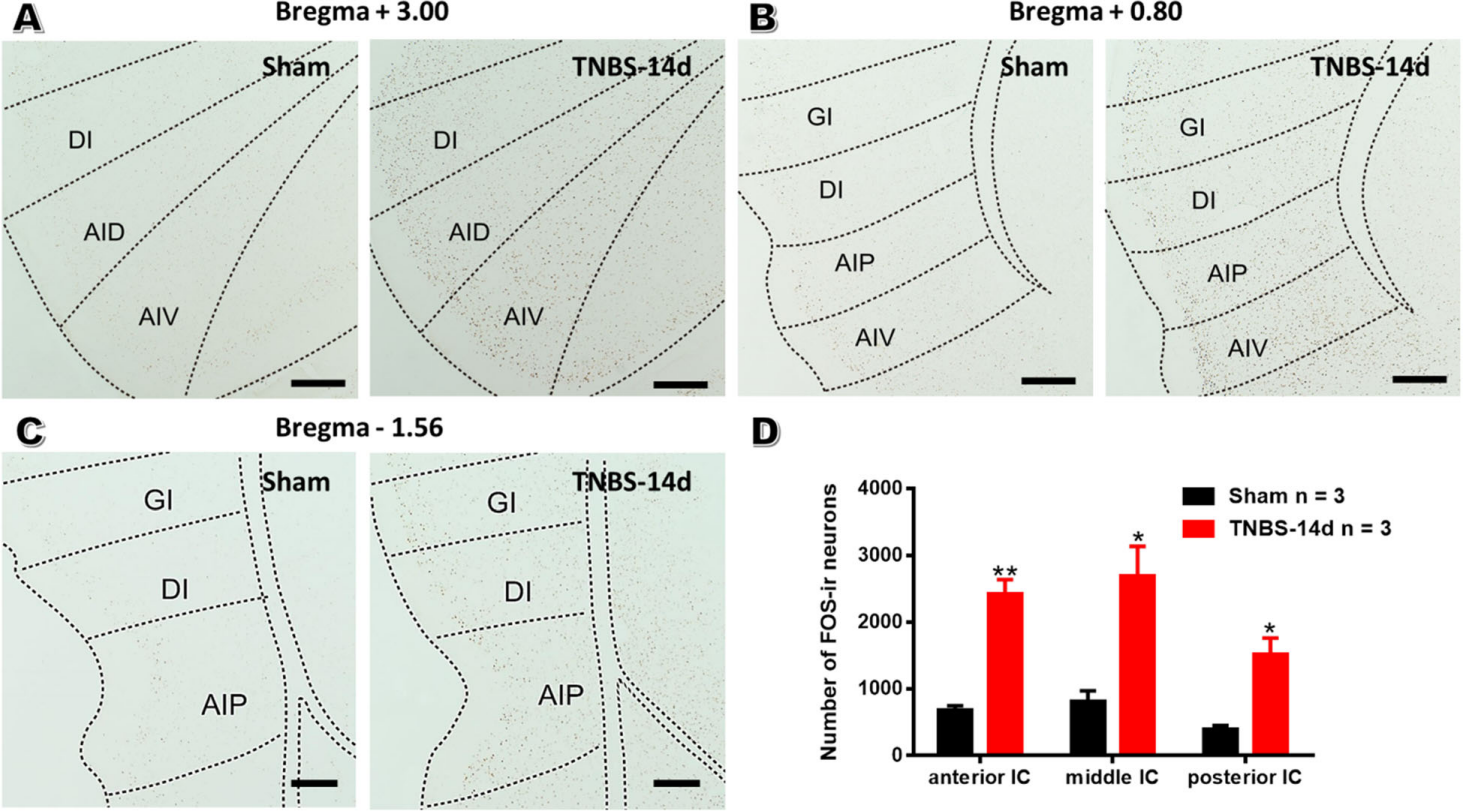
The number of FOS-expressing neurons is up-regulated within IC in CP rats. **a-c**: Immunochemical staining of FOS within different coronal sections of IC in sham (left) and CP rats on POD 14 (right). Bar = 200 μm. **d**: Histogram showing the qualification of FOS-expressing neurons within different parts of IC in saline or TNBS-treated rats. *n* = 3 slices from 3 rats in each group, unpaired *t*-test, * *P* < 0.05, ** *P* < 0.01, TNBS vs sham

FOS is widely used as an indicator of neuronal activation under the condition of pain stimuli [37]. Our previous study indicated that IC exhibited increased number of FOS-expressing neurons in the IBS mouse model [32]. In the present study, FOS immunostaining was performed in brain sections containing IC at the time point of POD 14, when rats exhibited robust abdomen hyperalgesia and anxiety. As shown in Fig. 2a-c, TNBS treatment markedly increased insular FOS expression in both II-III and V-VI layers. Since IC is a long brain region along the rostro-caudal axis, we calculated the number of FOS-immunoreactive neurons in the anterior, middle and posterior parts of IC between sham and TNBS groups. Quantification data showed that TNBS treatment increased the number of FOS-expressing neurons within the whole IC, including anterior IC (TNBS: 2425 ± 211.99 vs saline: 679 ± 65.39; *P* < 0.01; *n* = 3 rats for each group), middle IC (TNBS: 2698.70 ± 441.55 vs saline: 806 ± 161.36; *P* < 0.05) and posterior IC (TNBS: 1517.30 ± 246.81 vs saline: 393 ± 60.02; *P* < 0.05) (Fig. 2d). These data provided evidence for the activation of IC under the condition of painful CP, laying morphological foundation for subsequent investigations of electrophysiological changes.

### Insular post-LTP is occluded after TNBS treatment

**Fig. 3:**
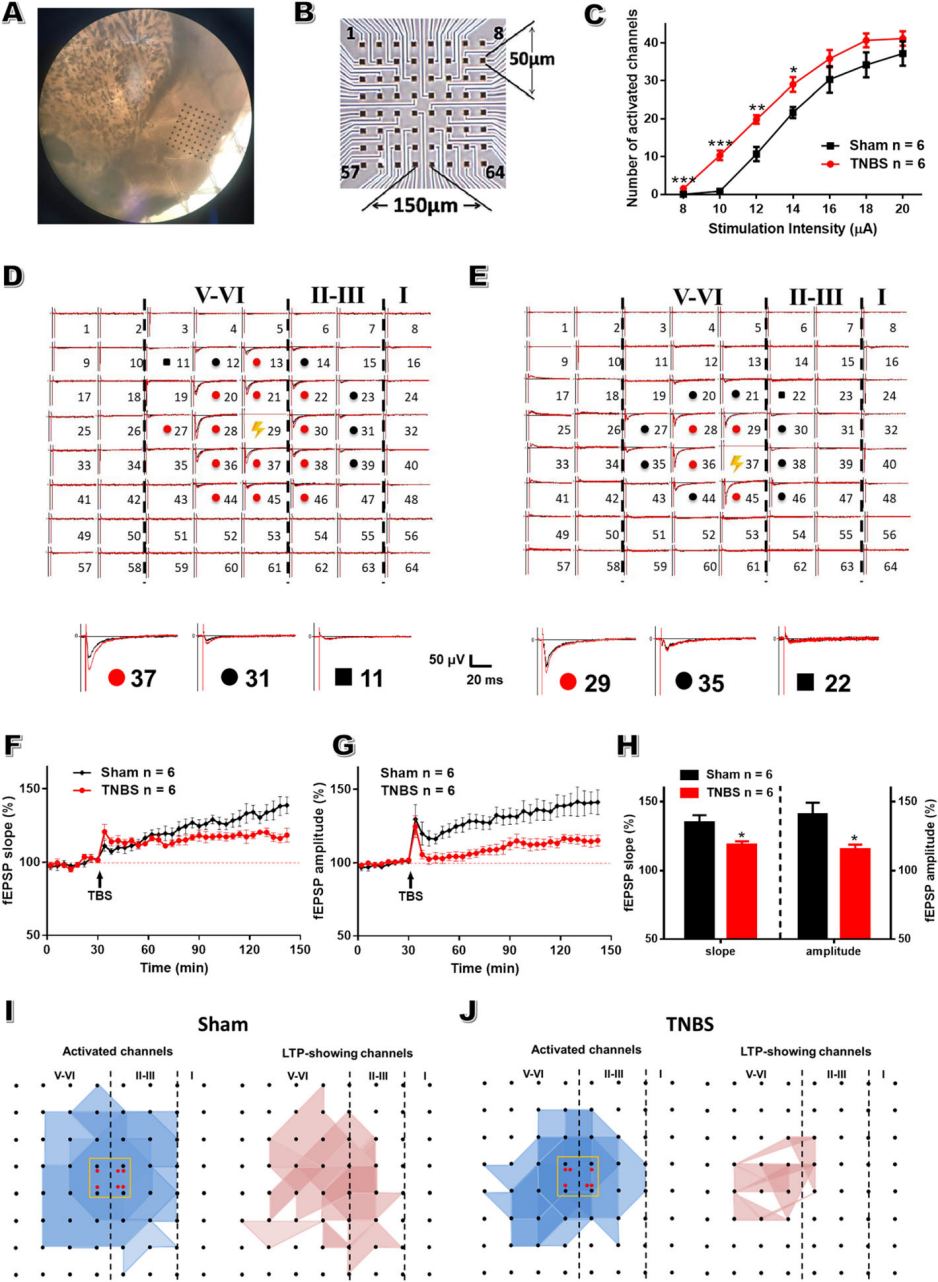
Post-LTP is occluded within aIC in rats with CP. **a**: Light microscopy photograph showing relative location of aIC within the probe. **b**: Schematic diagram showing the recording array arrangement. **c**: The input-output curve of the number of activated channels in slices of sham and CP rats, one-way repeated ANOVA. **d**, **e**: A sample of an overview of multisite synaptic responses recorded at baseline (black) and 2 h after TBS (red) in sham and CP groups, respectively. The flash denotes the stimulated channel. Red and black filled circles mark all activated channels undergoing and not undergoing LTP, respectively, while the black rectangle represents a typical channel not exhibiting any response in the baseline. These example traces are shown in an enlarged scale below. Vertical lines demarcate different layers. **f, g**: Time course of averaged fEPSP slope and amplitude of all active channels in sham and TNBS groups. The arrow indicates the time of TBS application. Dashed line indicates the baseline of 100% slope or amplitude. **h**: The average slope and amplitude of fEPSPs of all active channels within the last 20 min of 140 min recording in sham and TNBS groups, unpaired *t*-test. **i**, **j**: The polygonal diagram of activated (blue) and LTP-occurring (red) channels within aIC after TBS in sham (**i**) and TNBS (**j**) groups. The red dots indicate the stimulation sites. *n* = 6 slices from 6 rats in each group. * *P* < 0.05, ** *P* < 0.01, *** *P* < 0.001, TNBS vs sham

The advantage of multi-electrode array recordings is to record multiple sites simultaneously, making it possible to examine insular neuroplastic changes in both space and time dimensions [35]. In subsequent studies, we concentrated on the anterior part of IC since it exhibited more obvious increase in FOS-expressing neurons in CP rats than other parts of IC. Moreover, aIC participates in both sensory and emotional aspects of chronic pain [28, 29]. We recorded sections containing aIC at the level of the corpus callosum connection ranging from bregma + 2.58 ~ + 1.68 mm according to the rat atlas in stereotaxic coordinates [38]. The relative location of MED64 probe within the section was shown in Fig. 3a, b. Firstly, the input (stimulation intensity)-output (the number of activated channels) curve markedly shifted to the left in CP rats compared to sham group on POD 14 (Fig. 3c), indicating the hyperactivity of local insular neural network in CP rats. Secondly, as shown in Fig. 3d, we successfully induced post-LTP in both superficial and deeper layers within aIC after a TBS protocol applied in the deeper layer of aIC in sham rats. However, this potentiation was markedly reduced in the slices of CP rats (Fig. 3d-h, slope: 134.70 ± 5.32% of baseline in CP rats vs 118.50 ± 2.65% in sham rats; amplitude: 140.8 ± 8.37% in CP rats vs 115.40 ± 3.46% in sham rats; *P* < 0.05; *n* = 6 slices from 6 rats in each group). Finally, spatial analysis of post-LTP distribution indicated that CP rats displayed an obvious shrinkage of LTP map compared with sham group (Fig. 3i-j). This occlusive effect denotes that TNBS-triggered plasticity shares similar mechanisms with that of electrically-induced LTP.

### Enhanced neuronal activity within aIC of CP rats

**Fig. 4:**
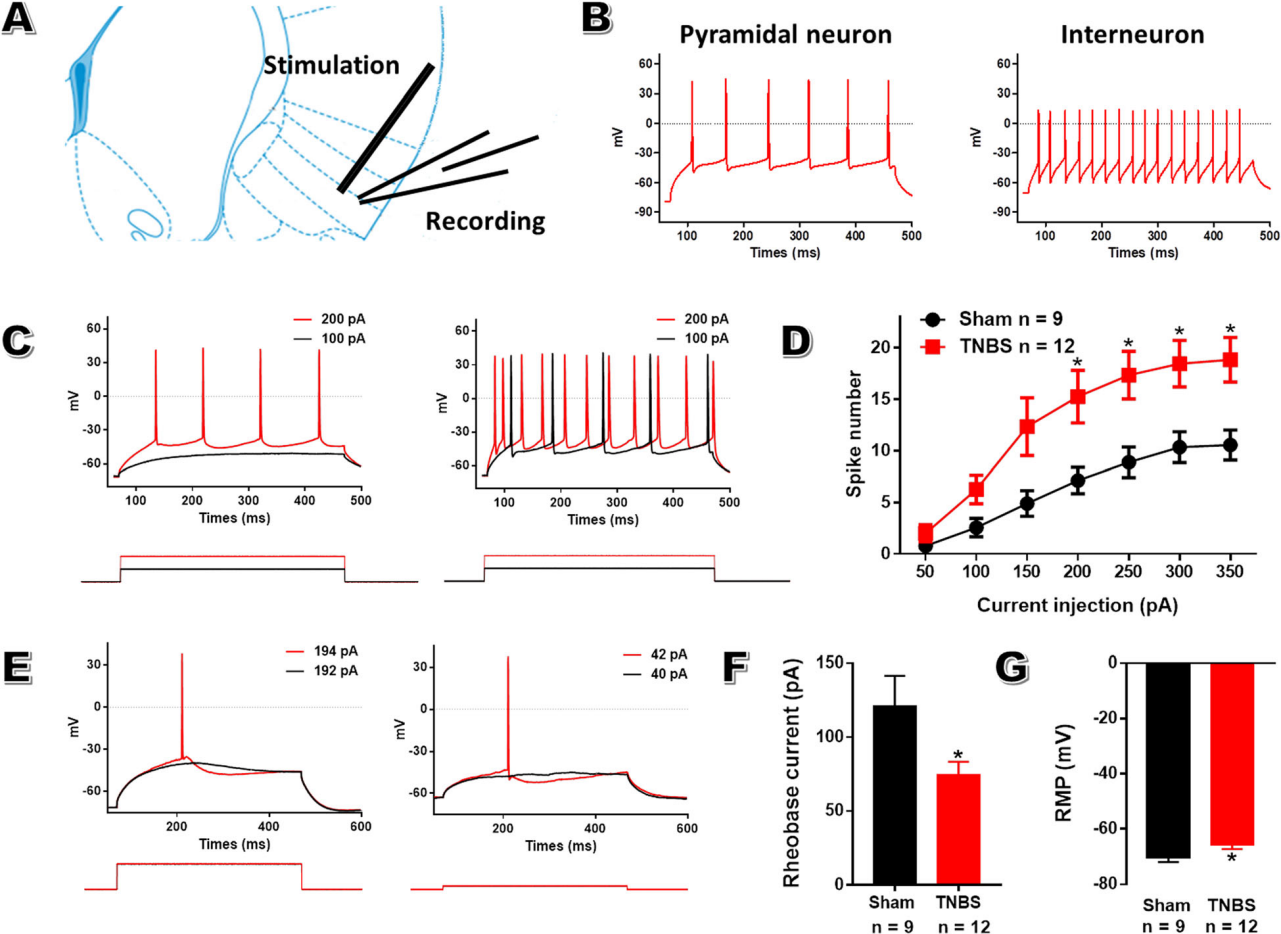
Enhanced pyramidal neuron excitability within aIC after TNBS treatment. **a**: Schematic diagram indicating the placement of stimulating and recording electrodes in the aIC. **b**: The different firing patterns of pyramidal neuron (left, repetitive action potentials with frequency adaptation) and interneuron (right, fast-spike firing) within aIC after positive current injected into the cell under current-clamp mode. **c**: Sample traces of the spikes recorded in pyramidal neurons within the superficial layer of aIC of sham (left) and CP (right) rats in response to depolarizing current injections, step = 100 pA, duration = 400 ms. **d**: The spike number input-output curve from CP rats was steeper than that from sham rats, one-way repeated ANOVA. **e**: Sample traces showing CP rats exhibited decreased rheobase current (42 pA in CP rats vs 194 pA in sham rats) and hyperpolarized RMP (− 63.30 mV in CP rats vs − 71.22 mV in sham rats) in insular pyramidal neurons, step = 2 pA, duration = 400 ms. **f**: The rheobase current was decreased in CP rats (74.33 ± 8.97 pA) compared to sham rats (120.7 ± 20.85 pA), unpaired *t*-test. **g**: The RMP was increased in CP rats (− 70.3 ± 1.57 mV) compared to sham rats (− 65.67 ± 1.48 mV), unpaired *t*-test. *n* = 9 in sham group and 12 in TNBS group, * *P* < 0.05, TNBS vs sham

Then, we assessed electrophysiological changes of individual insular pyramidal neurons in layers II-III, which receive sensory inputs from cortical or subcortical areas, via whole-cell patch-clamp recordings on POD 14 after TNBS treatment (Fig. 4a). Pyramidal neurons were identified via spike frequency adaptation in response to prolonged depolarizing current injection (Fig. 4b). Firstly, we investigated the changes of neuronal activity under current-clamp mode. The input (the intensity of injection current)-output (spike number) curve obviously shifted to the left in CP rats compared to sham rats (Fig. 4c, d). In addition, pyramidal neurons in CP rats showed reduced rheobase current and decreased resting membrane potential (Fig. 4e-g, Table 2). All these suggest the hyperexcitability of insular pyramidal neurons in CP rats.

**Table 2:** Summary of membrane properties of insular pyramidal neurons in sham and CP rats

	Sham group	CP group	*P*
Passive membrane properties			
Resting potential (mV)	−70.30 ± 1.48	−65.67 ± 1.42	0.048
Membrane resistance (MΩ)	225.8 ± 30.84	201.33 ± 22.35	n.s.
Access resistance (MΩ)	23.44 ± 1.34	23.13 ± 2.00	n.s.
Membrane time constant (ms)	2.07 ± 0.26	1.89 ± 0.25	n.s.
Active membrane properties			
Current threshold (pA)	120.67 ± 19.65	74.33 ± 8.60	0.038
Voltage threshold (mV)	−38.54 ± 1.17	−39.45 ± 1.32	n.s.
AP amplitude (mV)	82.91 ± 2.31	83.84 ± 1.27	n.s.
AP rise slope (mV/ms)	132.90 ± 4.79	123.22 ± 5.15	n.s.
AP decay slope (mV/ms)	−46.00 ± 3.83	− 61.29 ± 4.11	0.021
AP latency (ms)	219.92 ± 41.45	384.42 ± 85.92	n.s.
Cell capacitance (pF)	82.81 ± 9.72	69.75 ± 7.11	n.s.

Values are means ± SEM. The level of significance was determined using Student’s unpaired t test. *P* values for the comparison of insular pyramidal neurons in sham and CP rats

### Enhanced presynaptic transmitter release probability within aIC of CP rats

**Fig. 5:**
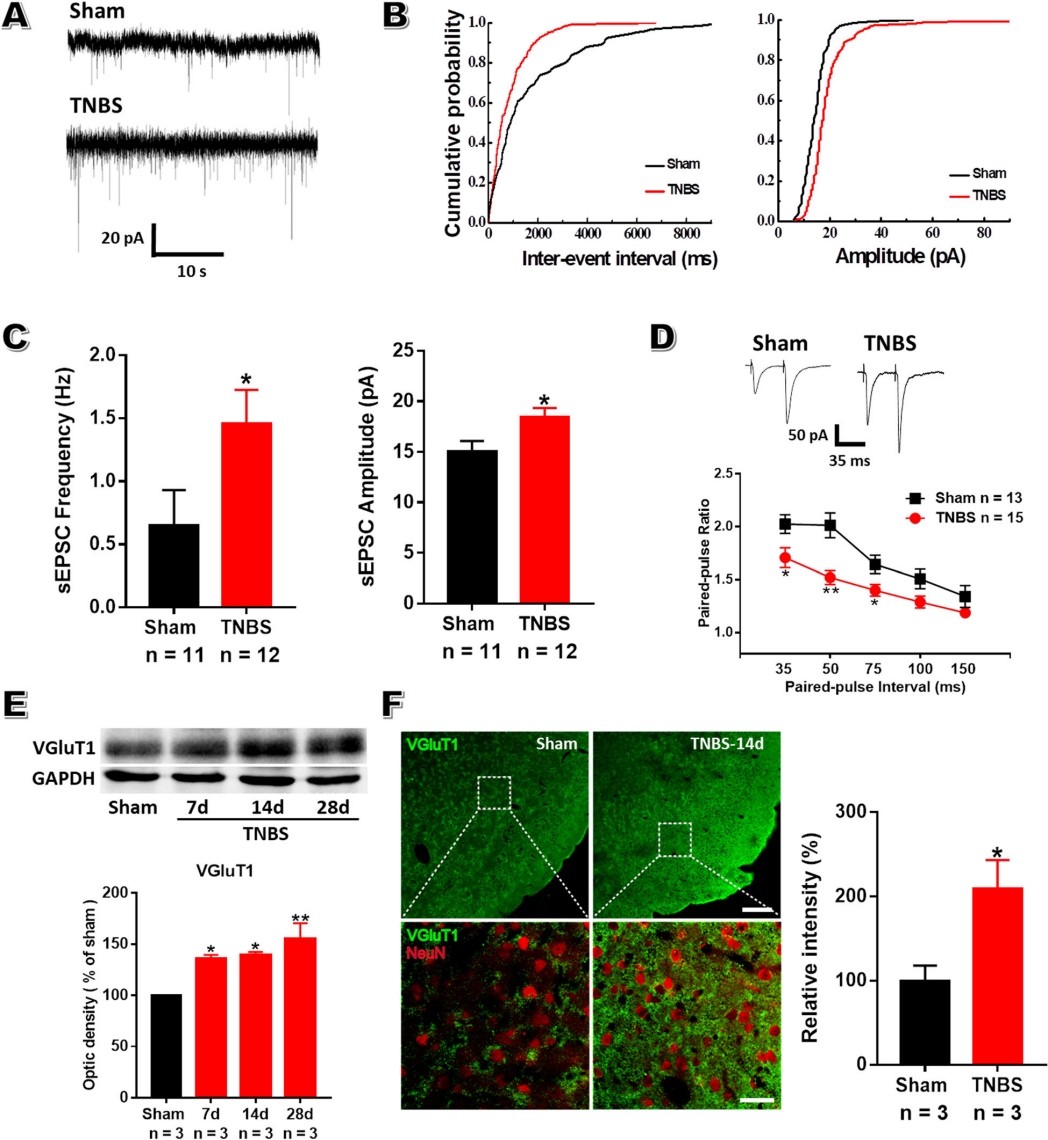
Enhanced presynaptic glutamate release within aIC after TNBS treatment. **a**: Representative sEPSCs recorded in the superficial layer of aIC from sham (top) and TNBS-treated (bottom) rats holding at − 60 mV. **b**: Cumulative inter-event interval (left) and amplitude (right) histograms of sEPSCs recorded in the same neurons showed in (**a**). **c**: Summary plots showing the frequency (left) and the amplitude (right) of sEPSC was increased in CP rats (1.46 ± 0.28 Hz, 18.48 ± 0.84 pA, *n* = 12) compared to sham rats (0.65 ± 0.26 Hz, 15.06 ± 0.96 pA, *n* = 11), unpaired *t*-test. **d**: Representative traces of PPF with an interval of 35 ms recorded in the superficial layer of aIC (top). PPF at time intervals of 35, 50 and 75 ms was reduced in CP rats (bottom). *n* = 13 cells in sham group and 15 in TNBS group, one-way repeated ANOVA. **e**: Representative western blot sample for VGluT1 within aIC obtained on POD 7, 14 and 28 (top). Statistical analyses showing enhanced expression of VGluT1 from POD 7 to 28 after TNBS treatment (bottom). *n* = 3 rats in each group, one-way ANOVA. **f**: The immunoreactivities of VGluT1 within aIC were remarkably increased in TNBS-treated rats compared to sham rats. Microphotographs indicating double-immunoflurescence histochemistry for VGluT1 (green) and NeuN (red) within aIC (left). The framed areas in upper images were magnified in lower images. Bars = 200 μm in upper images and 40 μm in lower images. Quantifications and statistical analyses of VGluT1 immunoreactivities presented in the graph (right). * *P* < 0.05, ** *P* < 0.01, TNBS vs sham

To explore whether there exist any changes in basal synaptic transmission within aIC after TNBS treatment, sEPSCs were recorded under voltage-clamp mode. A robust augmentation in both frequency and amplitude was observed in CP rats (Fig. 5a-c, frequency: 1.46 ± 0.28 Hz in CP rats vs 0.65 ± 0.26 Hz in sham rats; amplitude: 18.48 ± 0.84 pA in CP rats vs 15.06 ± 0.96 pA in sham rats; *P* < 0.05; *n* = 11 cells in CP group and 12 in sham group). These results suggest that enhanced presynaptic glutamate release and postsynaptic responsiveness both likely contribute to the enhanced excitatory synaptic transmission in the aIC of CP rats.

To verify that presynaptic mechanisms mediate the enhanced excitatory synaptic transmission in aIC, PPF, a measure of presynaptic function in which the response to the second stimulus is enhanced as a result of residual calcium in the presynaptic terminal after the first stimulus [39], was examined by electrical stimulation of the deeper layer of aIC. As seen in Fig. 5d, PPF was significantly reduced at time intervals of 35, 50 and 75 ms in the aIC of CP rats compared to sham rats.

VGluT1 is necessary for excitatory synaptic transmission through glutamate packaging and exocytosis within cerebral cortex, the number of which has a major impact on quantal size in glutamatergic neurons [40]. To examine if there are expressional alterations of VGluT1 within aIC in CP rats, biochemical and morphological studies were performed. Biochemical results showed up-regulated expression of VGluT1 along the course of CP (Fig. 5e), which was confirmed by immunohistochemical staining data showing more VGluT1-ir terminals were encountered in TNBS-treated rats on POD 14 than sham rats (Fig. 5f). These data suggest CP stimulates glutamate production and release, providing presynaptic evidence for enhanced synaptic transmission within aIC.

### Enhanced postsynaptic responsiveness of AMPAR and NMDAR within aIC of CP rats

**Fig. 6:**
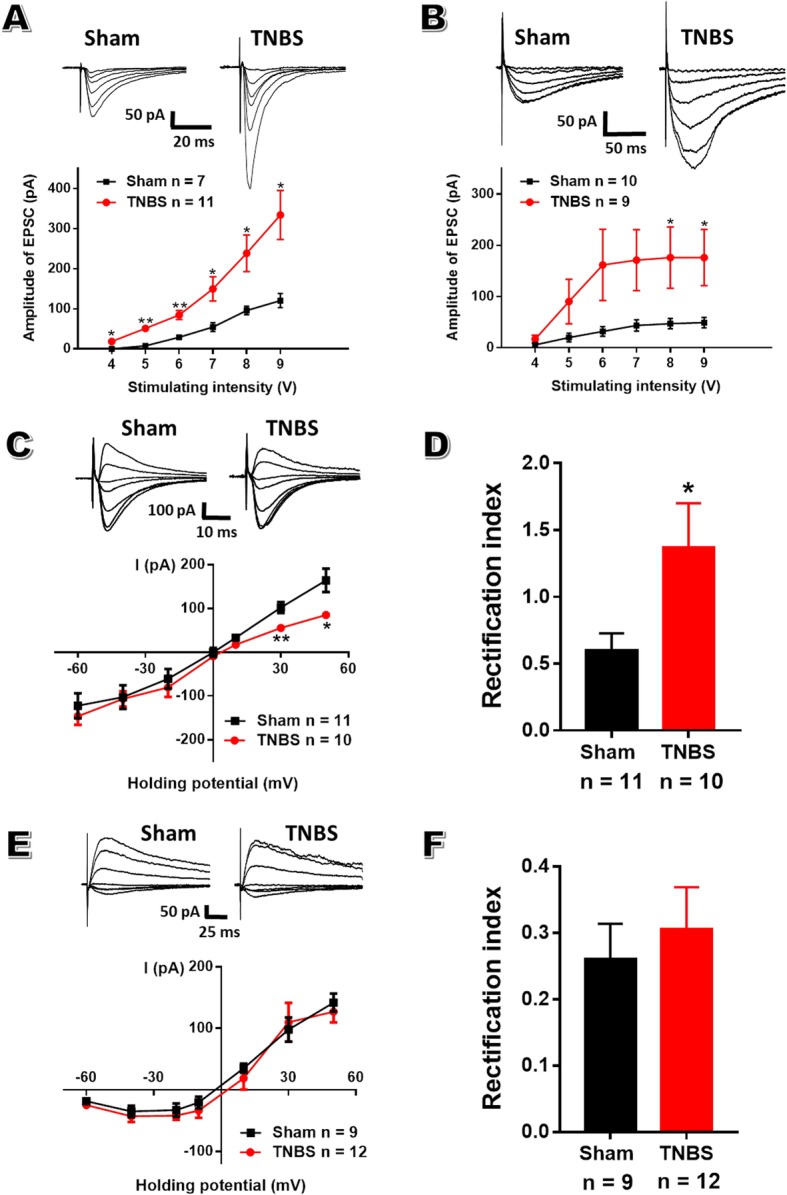
Enhanced AMPAR and NMDAR currents within aIC after TNBS treatment. **a**: The AMPAR-mediated synaptic input-output curve of CP rats (*n* = 11) was steeper than that from sham rats (*n* = 7), one-way repeated ANOVA. **b**: The NMDAR-mediated synaptic input-output curve of CP rats (*n* = 9) was steeper than that from sham rats (*n* = 10), one-way repeated ANOVA. **c**: *I-V* curves of AMPAR-EPSCs of aIC pyramidal neurons recorded at holding potentials ranging from − 60 to + 50 mV in sham and CP rats, one-way repeated ANOVA. **d**: Comparison of the rectification index of AMPAR-EPSCs in sham (*n* = 11) and CP (*n* = 10) rats, unpaired *t*-test. **e**: *I-V* curves of NMDAR-EPSCs of aIC pyramidal neurons recorded at holding potentials ranging from − 60 to + 50 mV in sham and CP rats, one-way repeated ANOVA. **f**: Comparison of the rectification index of NMDAR-EPSCs in sham (*n* = 9) and CP (*n* = 12) rats, unpaired *t*-test. * *P* < 0.05, ** *P* < 0.01, TNBS vs sham

To explore postsynaptic mechanisms underlying the enhanced excitatory synaptic transmission in the aIC of CP rats, we isolated AMPAR and NMDAR-mediated EPSCs in the presence of AP-5 and CNQX, respectively. Input (stimulation intensity) - output (EPSC amplitude) curves of both AMPAR (Fig. 6a) and NMDAR (Fig. 6b) -mediated currents were significantly shifted to the left after TNBS treatment, suggesting that both AMPAR and NMDAR-related mechanisms participate in the postsynaptic enhancement of aIC during painful CP.

AMPARs channels are composed of four subunits. GluR2-containingAMPARs are impermeable to Ca^2+^ [41] while GluR2-lackingAMPARs possess high Ca^2+^permeability and show inward-rectifying currents [42]. To determine whether TNBS treatment alters the prevalence of GluR2-lacking and GluR2-containing AMPARs within aIC, we examined current-voltage relationship of AMPAR-EPSCs of insular pyramidal neurons. In sham rats, the AMPAR-EPSCs showed a near-linear *I-V* relationship with a reversal potential near 0 mV, while the amplitude of AMPAR-EPSCs of CP rats was reduced at positive membrane potentials (Fig. 6c). The rectification index (I_-40mV_/I_+50mV_) of AMPAR-EPSCs was significantly increased in CP rats compared with sham rats (1.37 ± 0.31 in CP rats vs 0.60 ± 0.12 in sham rats; *n* = 11 neurons in CP rats and 10 in sham rats; *P* < 0.05; Fig. 6d). All these data suggest chronic pancreatitis increases GluA2-lacking AMPAR prevalence in aIC pyramidal neurons. Moreover, the *I-V* relationship of NMDAR-mediated EPSCs from sham group showed a typical outward rectification, which was similar to that of CP rats (Fig. 6e, f), suggesting no change in the characteristic of NMDAR-mediated currents during painful CP.

### Enhanced phosphorylation and membrane accumulation ofNR2B and GluR1 within aIC in CP rats

**Fig. 7:**
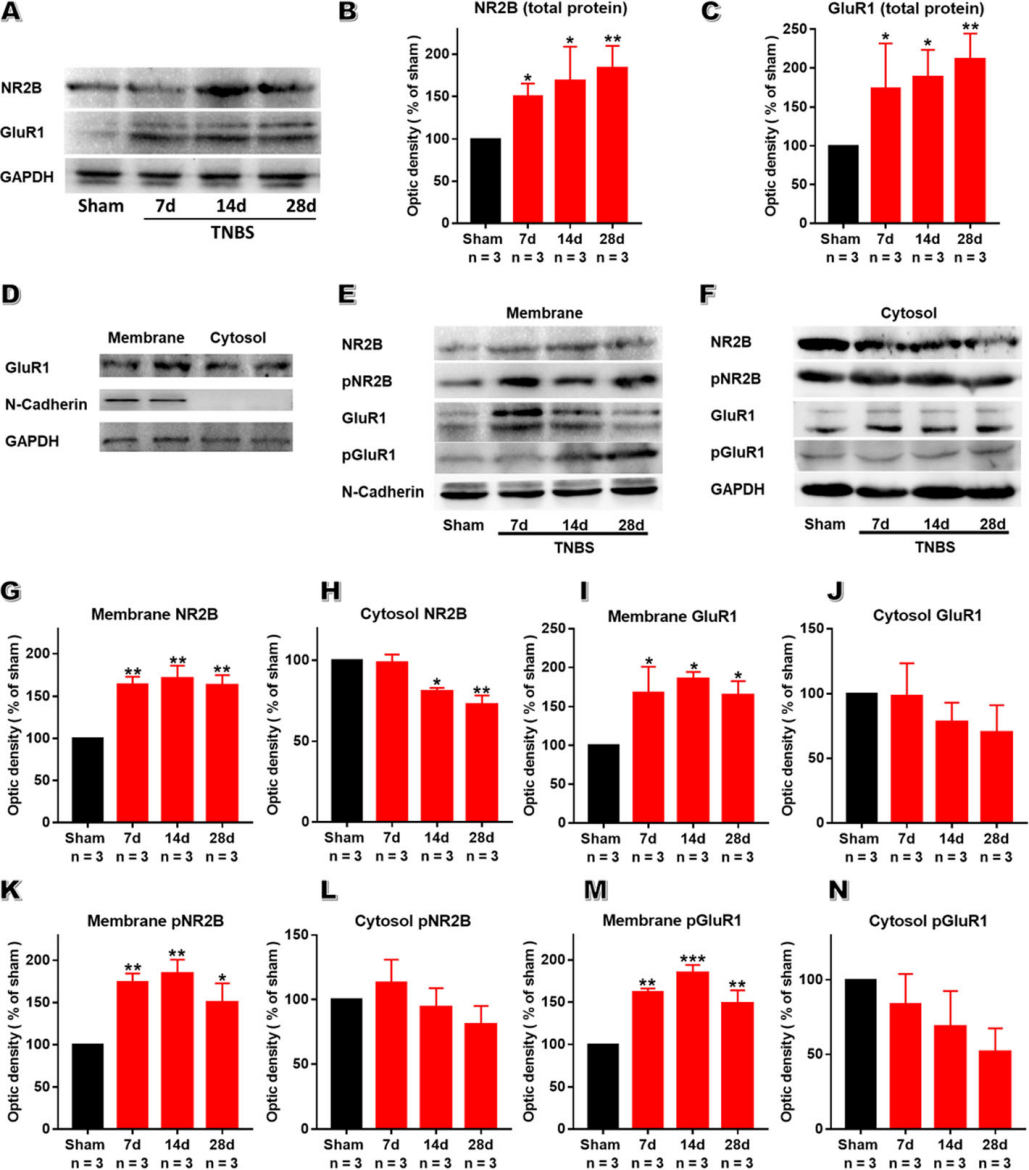
TNBS treatment facilitates the phosphorylation and the trafficking of insular glutamate receptor subunits into membrane. **a**: Representative western blot for NR2B and GluR1 within aIC on POD 7, 14 and 28. **b**, **c**: The expressions of NR2B (**b**) and GluR1 (**c**) were significantly enhanced from POD 7 to 28 after TNBS treatment. **d**: Fractionation of insular tissue was probed for N-cadherin and GAPDH to verify the accuracy of subcellular fractionation procedure. **e, f**: Representative western blot samples for membrane (**e**) and cytosol (**f**) NR2B, pNR2B, GluR1 and pGluR1 within aIC obtained on POD 7, 14 and 28 in TNBS-treated rats and sham rats. **g, h** Membrane NR2B was significantly increased on POD 7, 14 and 28 after TNBS treatment compared to sham group while cytosol NR2B was significantly decreased on POD 14 and 28. **i**, **j** Membrane GluR1 was significantly increased on POD 7, 14 and 28 after TNBS treatment compared to sham group while cytosol GluR1 showed no change. **k**, **l** Membrane pNR2B was significantly increased on POD 7, 14 and 28 after TNBS treatment compared to sham group while cytosol NR2B showed no change. **m**, **n** Membrane pGluR1 was significantly increased on POD 7, 14 and 28 after TNBS treatment compared to sham group while cytosol pGluR1 showed no change. *n* = 3 rats in each group, one-way ANOVA, * *P* < 0.05, ** *P* < 0.01, *** *P* < 0.001, TNBS vs sham

NMDAR channels are composed of two major subunits, including NR1 and NR2A-D. Among these, NR2B is the predominant subunits within IC, which determines the trafficking and synaptic localization of NMDAR and plays an important role in chronic neuropathic pain [15, 43]. As the key subunit of AMPAR (including GluR1–4 subunits) within IC, GluR1 generates AMPAR trafficking and integration within synaptic membranes and contributes to the chronification of neuropathic pain [14, 44]. Then, we examined the expressional changes of these two representative glutamate receptor subunits, NR2B and GluR1, in CP rats. In consistent with our functional evidence, biochemical analysis showed that CP triggers long-term increase in the expression of AMPAR and NMDAR within aIC (Fig. 7a-c). All these data suggest both insular AMPAR and NMDAR are involved in postsynaptic enhancement during painful CP.

Both AMPAR and NMDAR are dynamically shifting in and out of membrane according to synaptic activity, which in turn determines synaptic strength [45, 46]. To determine whether membrane glutamate receptors increased in CP rats, biochemical analyses were performed to measure the abundance of NR2B and GluR1 in both membrane and cytoplasmic fractions. A clear isolation of membrane and cytoplasmic protein was confirmed by the distribution of N-Cadherin, a neural membrane marker, in membrane protein instead of its cytoplasmic counterpart (Fig. 7d). Biochemical analysis of NR2B within aIC at different time points showed membrane expression of NR2B was robustly up-regulated (Fig. 7e and g), while its cytoplasmic content was significantly down-regulated in CP rats as compared with sham group (Fig. 7f and h). In addition, the abundance of membrane GluR1 was also markedly heightened in CP rats (Fig. 7e and i), with no striking change in that of cytoplasmic GluR1 (Fig. 7f and j). In line with increased inward rectification of AMPAR-mediated currents mentioned before, these data supports the increase of membrane calcium-permeable AMPARs in painful CP rats.

Phosphorylation is an important posttranslational modification for glutamateric receptors during their membrane targeting [43]. Phosphorylation of NMDAR, especially tyrosine phosphorylation of NR2B at Tyr^1472^ site, leads to enhanced synaptic NMPARs via inhibiting NMDAR endocytosis and mediates the chronification of neuropathic pain [15]. Likewise, serine phosphorylation of GluR1 at Ser^845^ site also contributes to the localization and function of insular AMPAR in mice with nerve injury [14]. To confirm whether similar posttranslational modifications occurred under the condition of painful CP, we measured the phosphorylation status of membrane NR2B Tyr^1472^ site and GluR1 at Ser^845^ site using phosphorylation site specific antibodies. Membrane expression of pNR2B at Tyr^1472^ site was robustly ramped up along the course of CP (Fig. 7e and k), while no striking difference was detected in its cytoplasmic counterpart between sham and CP rats (Fig. 7f and l). Furthermore, the content of membrane pGluR1 at Ser^845^ site also increased along the course of CP (Fig. 7e and m), while cytoplasmic pGluR1 remained unchanged, albeit a tendency of declined expression of cytoplasmic pGluR1 in CP group (Fig. 7f and n). All these demonstrated TNBS injection leads to the recruitment and modification of glutamate receptors, providing postsynaptic evidence for the enhanced synaptic transmission within aIC.

### Inhibiting excitatory synaptic transmission within aIC relieves abdomen hyperalgesia

**Fig. 8:**
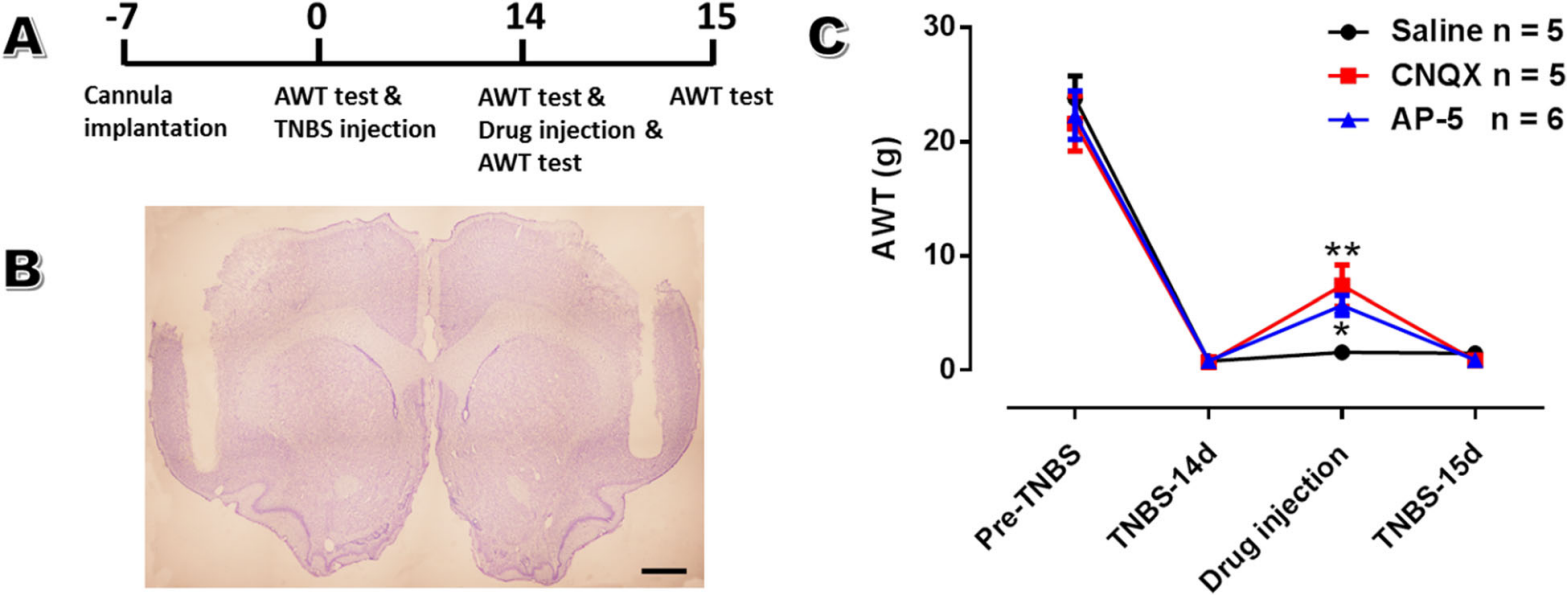
Bilateral microinjection of CNQX and AP-5 within aIC alleviates visceral hypersensitivity of CP rats. **a**: The schematic diagram of the behavioral experiment. **b**: A representative coronal section showing the sites of cannula implantation within bilateral aIC. Bar = 1 mm. **c**: Bilateral microinjections of CNQX and AP-5, instead of saline, significantly enhanced the AWT in CP rats on POD 14. *n* = 5 in saline and CNQX-treated groups and 6 in AP5-treated group, one-way ANOVA, * *P* < 0.05, ** *P* < 0.01, CNQX or AP5 vs saline

To determine whether the enhanced excitatory transmission within aIC is detrimental in the development of pancreatitis pain, the effects of blocking insular glutamatergic transmission via CNQX and AP-5 on behavioral manifestations of pancreatic nociception were determined (Fig. 8a, b). Bilateral microinjection of CNQX and AP-5 into aIC reversed abdomen hyperalgesia in CP rats on POD 14 (AWT: 7.40 ± 1.82 g for CNQX group, 5.67 ± 0.87 g for AP-5 group and 1.56 ± 0.56 g for saline group; *n* = 6 in AP-5 group and 5 in both CNQX and saline groups; *P* < 0.05; Fig. 8c), which returned to baseline on POD 15 (0.84 ± 0.26 for CNQX group, 0.90 ± 0.22 for AP-5 group and 1.48 ± 0.30 for saline group; *P* > 0.05; Fig. 8c). These behavioral results indicated the excitatory glutamatergic transmission within aIC contributes to hyperalgesia of CP rats.

### Inhibiting excitatory aIC neurons relieves abdomen hyperalgesia and anxiety

**Fig. 9:**
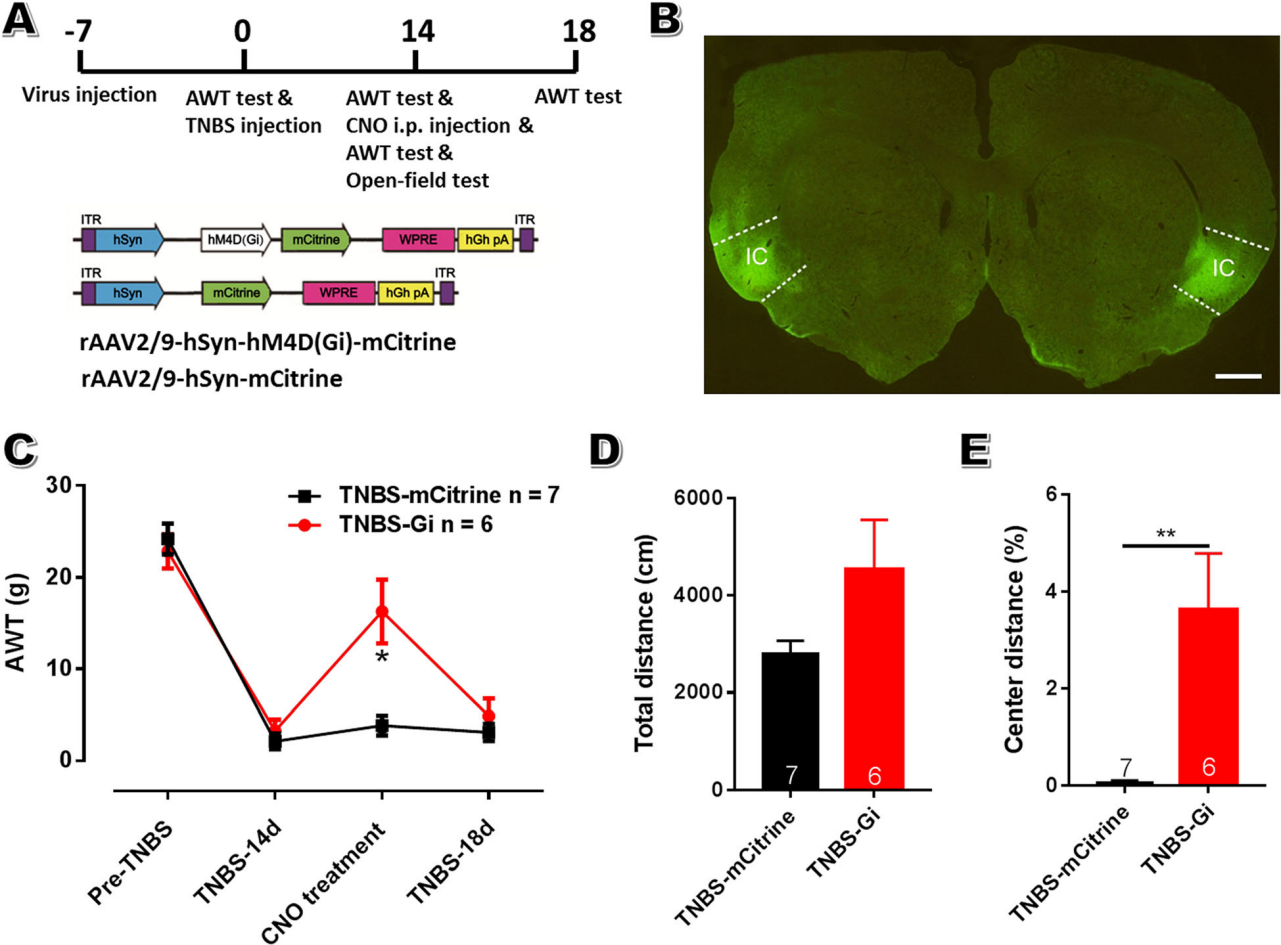
Non-selectively chemogenetic inactivation of bilateral insular neurons alleviates abdomen mechanical hyperalgesia and anxiety-like behavior in CP rats. **a**: The schematic diagram of the behavioral experiment (up) and rAAV2/9-hSyn-hM4Di-mCitrine construct (down). **b**: A representative coronal section showing virus injection sites within bilateral aIC. Bar = 1 mm. **c**: Inhibiting bilateral insular neurons via i.p. CNO injection significantly increased the AWT in CP rats on POD 14. **d**, **e**: Inhibiting bilateral insular neurons led to a trend toward increased total traveling distance (**d**) and a significant increase in central traveling distance (**e**) in CP rats. *n* = 7 in mCitrine group and 6 in Gi group, unpaired *t*-test. * *P* < 0.05, ** *P* < 0.01, Gi vs mCitrine

Previous studies have substantiated that insular ablation could alleviate both neuropathic pain and stress-related visceral pain [16, 17, 23]. In this study, we utilized chemogenetics to reversibly, non-specifically inhibit the activity of aIC neurons in CP rats. We targeted inhibitory designer receptor exclusively activated by designer drug (hM4Di DREADD) [47] to insular neurons by injecting recombinant adeno-associated vectors rAAV2/9-hSyn-hM4Di-mCitrine into aIC of CP rats (Fig. 9a, b). Behavioral results showed that non-selectively inhibiting insular neurons alleviated abdomen hyperalgesia of CP rats on POD 14 (AWT: 16.29 ± 3.49 g in group and 3.86 ± 1.10 g in mCitrine group; *n* = 6 rats in Gi group and 7 in mCitrine group; *P* < 0.05; Fig. 9c). No significant difference in baseline AWT was seen 3 days later after CNO treatment between these groups. To determine whether aIC is necessary for the resultant hypolocomotion and anxiety-like behavior, open field test was subsequently performed. Comparing to control group, bilateral insular inactivation did not influence animal locomotion in the open field, albeit an increasing tendency seen in Gi group (Fig. 9d). However, bilateral insular inactivation alleviated the decreased exploratory behavior of CP rats on POD 14 (Center distance %: 3.63 ± 1.06 in Gi group and 0.28 ± 0.11 g in mCitrine group; *P* < 0.01; Fig. 9e). All these suggest that aIC mediates hyperalgesia and pain-related anxiety in CP rats.

**Fig. 10:**
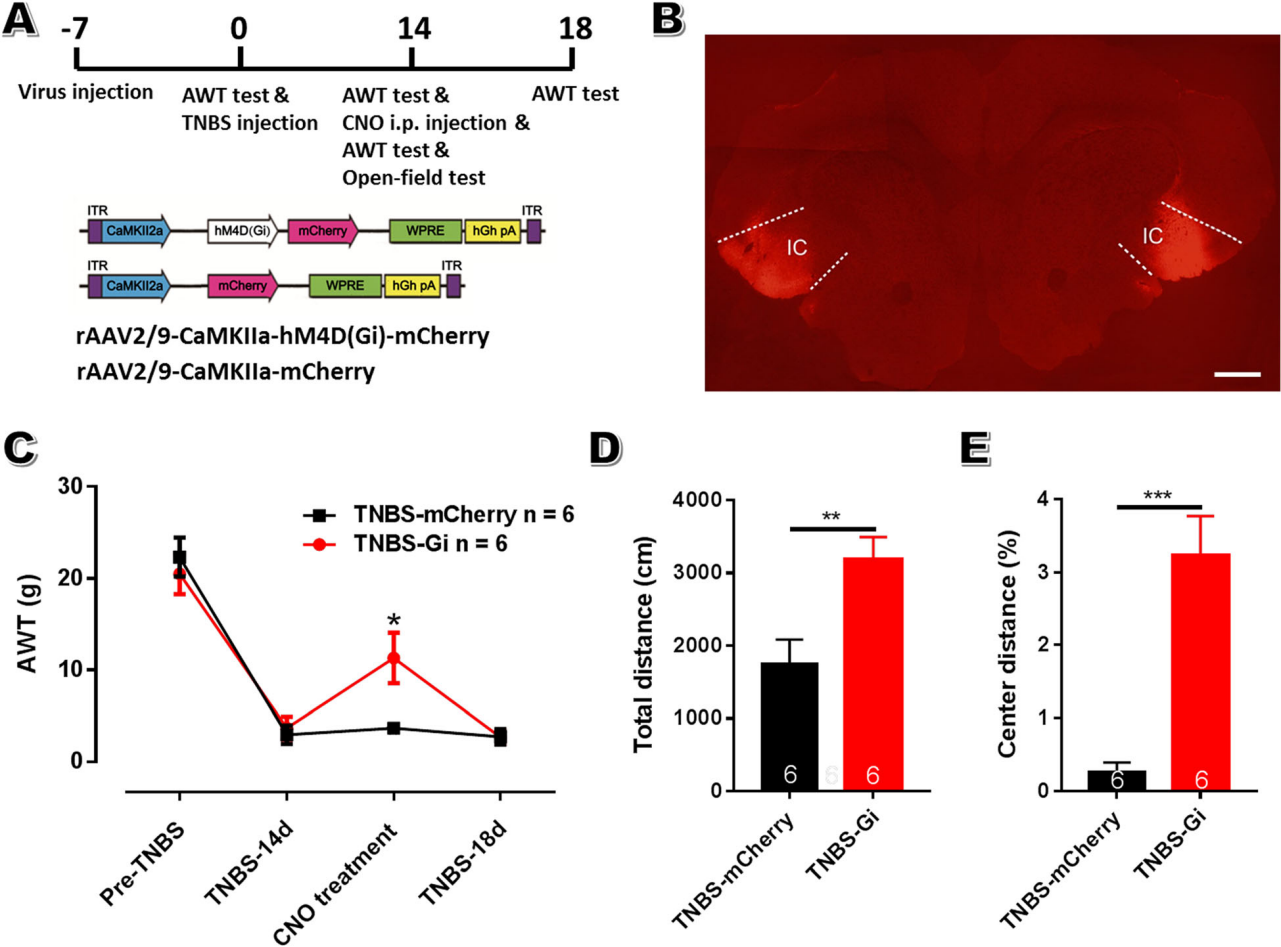
Chemogenetic inactivation of bilateral insular pyramidal neurons alleviates abdomen mechanical hyperalgesia and anxiety-like behavior in CP rats. **a**: The schematic diagram of the behavioral experiment (up) and rAAV2/9-CaMKIIa-hM4Di-mCherry construct (down). **b**: A representative coronal section showing virus injection sites within bilateral aIC. Bar = 1 mm. **c**: Inhibiting bilateral insular pyramidal neurons via i.p. CNO injection significantly increased the AWT in CP rats on POD 14. **d**, **e**: Inhibiting bilateral insular neurons led to a significant increase in total traveling distance (**d**) as well as central traveling distance (**e**) in CP rats. *n* = 6 in each group, unpaired *t*-test. * *P* < 0.05, ** *P* < 0.01, *** *P* < 0.001, Gi vs mCherry

To verify whether the pain-facilitating role of aIC was mediated by excitatory pyramidal neurons, we used DREADDs to specifically suppress the activity of bilateral IC pyramidal neurons via CaMKIIa promoter-driven expression using rAAV vectors (rAAV2/9-CaMKIIa-hM4Di-mCherry) under the condition of painful CP (Fig. 10a, b). Inactivation of aIC excitatory neurons elicited robust analgesic effects in CP rats on POD 14 (AWT: 11.33 ± 2.77 g in Gi group and 3.67 ± 0.56 g in mCitrine group; *n* = 6 rats in each group; *P* < 0.05; Fig. 10c). Interestingly, inhibiting the activity of aIC pyramidal neurons also alleviated the decrease in traveling distance as well as the exploratory behavior of CP rats in open field test (Fig. 10d, e). All these indicate an important role of insular pyramidal neurons in the production of hyperalgesia and anxiety during the process of CP.

## Discussion

In this study, we provided morphological, biochemical, physiological and behavioral evidence for that the excitatory synaptic transmission within aIC undergoes long-term potentiation and contributes to behavior sensitization during painful CP. Both presynaptic and postsynaptic mechanisms (the enhanced glutamate release and the recruitment of membrane glutamate receptors) are involved in this process. In addition, we further revealed that anterior insular pyramidal neurons exhibit hyperactivity under the condition of painful CP. Chemogenetic inhibition of aIC pyramidal neurons alleviates both hyperalgesia and pain-related anxiety in CP rats.

### Insular cortex and visceral pain

Insular cortex plays an important role in the processing of somatic and visceral information, especially interoceptive signals from internal environment that reflects autonomic activity [26]. As a limbic integrating region, it is also considered critical for the generation of emotional feelings [27] and mood disorders, including anxiety and depression [48]. With regard to pain sensation, IC is a primary cerebral reception area for physiological visceral pain signals from the gut [49]. Accumulating imaging studies suggest IC also exhibits activity changes in chronic visceral pain states, such as IBS and bladder pain syndrome [50]. Under the condition of pancreatitis pain, what we know is that IC underwent both structural and functional reorganization [7–9, 11]. However, the specific role of IC during painful CP is not clear. Recent preclinical researches indicated that, similar with the condition of neuropathic pain [14, 15], the excitatory transmission within aIC was enhanced in IBS rat model [21, 22] and bilateral aIC lesion could markedly inhibit the formation of visceral hypersensitivity [23]. Here, we observed that chronic pancreatitis triggered long-term up-regulation of glutamatergic excitatory transmission and neural activity within the aIC, which may broaden our understanding of the role of IC in chronic visceral pain.

Chronic pain is frequently accompanied by affective disorders, which exacerbate the sensory abnormalities of chronic pain [51, 52]. We observed long-term anxiety in TNBS-treated rats demonstrated by hypolocomotion and reduced exploratory behavior in the open field, which was also seen in IBS-related visceral pain [32]. This hypolocomotion is not owing to motor coordination disorder since rota rod test showed no difference in the falling latency between sham and CP rats (unpublished observations). It is worth noting that decreased locomotion is seldom seen in neuropathic pain [51]. One parsimonious explanation is that visceral pain is usually more unbearable than somatic pain and causes severer emotional and autonomic disorders, which may lead to decreased motor desire. Considering these, both hypolocomotion and exploratory behavior might be important indicators for affective disorders in painful CP. IC has been supposed to participate in emotional pain modulation. Based on the connectivity profile, it is considered that aIC preferably mediates its emotional effect while pIC is more involved in its somatosensory feature [12]. This theory was supported by the fact that pIC lesion reduced mechanical allodynia instead of the depressive consequences of chronic neuropathic pain [28]. Our study showed that the inactivation of aIC alleviated behavioral hypolocomotion and anxiety in CP rats, revealing the critical role of aIC in encoding the affective aspect of chronic pain.

### Insular LTP and visceral pain

Excitatory synapses are highly plastic, and LTP of glutamatergic transmission within IC is a key cellular mechanism for pathological pain [13]. Inducing LTP-like neuroplastic changes via injecting NMDA into IC in naïve rats produces mechanical allodynia [53], while inhibiting insular LTP via PKM휁 inhibitor elicited analgesic effects under the condition of neuropathic pain [20]. In the present study, insular post-LTP could be induced via TBS stimulation in sham rats instead of TNBS-treated rats. This failure of insular post-LTP induction was also encountered in mice with neuropathic pain [15]. All these suggest that, as similar with neuropathic pain, chronic pancreatitis pain may also recruit similar molecular mechanisms with TBS-induced LTP.

Activity-dependent Ca^2+^ flux through NMDAR activation, induced by excessive presynaptic glutamate release, is esteemed to initiate downstream signaling events during insular post-LTP induction [13, 54]. Adenylate cyclase 1 (AC1) is one essential Ca^2+^-stimulated enzyme that converts ATP to cAMP and activates downstream signaling molecules, such as protein kinase A (PKA) and cAMP-response element-binding protein (CREB). Evidence obtained from neuropathic pain indicates that nerve injury promotes insular GluR1 phosphorylation and trafficking. This phosphorylation of synaptic GluR1 is site-specific since only phosphorylation at Ser^845^ site (a PKA site) instead of Ser^831^ site (a CaMKII and PKC site) was increased, suggesting the pivotal role of AC1-cAMP-PKA pathway under the condition of neuropathic pain [14]. Meanwhile, the activation of AC1-cAMP-PKA pathway also facilitates insular NR2B phosphorylation specifically at Tyr^1472^ (not Tyr^1336^ or Ser^1303^) and forms a positive feedback to enhance NMDAR function, thus mediating neuropathic pain after nerve injury [15].

In the present study, our electrophysiological data provided functional evidence for presynaptic and postsynaptic amplifications within aIC in CP rats, which was verified by subsequent biochemical studies indicating enhanced expression of VGluT1 as well as membrane NR2B and calcium-permeable GluR1 subunits. In addition, the amounts of pGluR1 at Ser^845^ and pNR2B at Tyr^1472^ also increased within aIC. Considering these, we proposed that excessive glutamate release induced by pancreatitis pain may trigger calcium influx into post-synaptic membrane and subsequent intracellular signaling pathways to phosphorylate GluA1-containing AMPA receptor and NR2B-containing NMDA receptor, which promotes membrane trafficking and long-term potentiation of insular excitatory transmission. During this process, AC1-cAMP-PKA pathway may play an important role in the modification and recruitment of glutamate receptors. These LTP-like neuroplastic changes are closely related to behavior allodynia since inhibiting insular plasticity via NMDAR and AMPAR antagonists relieved neuropathic pain [14, 15] as well as CP-related hyperalgesia in this study. These results provide novel cortical mechanisms for chronic visceral pain and shed light on drugs targeting at insular post-LTP, for example, the AC1 inhibitor NB001 [32] in the treatment of painful CP. One limitation of our study is that we did not examine whether other glutamate receptor subunits and phosphorylation sites are involved in painful CP. Detailed signaling mechanisms also warrant our further investigation.

Another limitation of this study is that we only observed the occlusive effects of insular post-LTP under the condition of painful CP. Apart from post-LTP, presynaptic LTP (pre-LTP) is a kind of NMDAR-independent LTP initially reported in hippocampus [55]. In 2015, this kind of LTP was detected within ACC by Koga et al. They further observed that this kind of LTP was occluded under the condition of chronic pain and anxiety, and erasing ACC pre-LTP exerted anxiolytic effects during chronic pain [56]. In light of these, pre-LTP is thought to mediates anxiety signals triggered by chronic pain, while post-LTP is related to behavioral hyperalgesia during chronic pain [57]. In a recent study, Zhuo M et al. succeeded in recording pre-LTP in the insular cortex, which shared similar molecular mechanisms with ACC pre-LTP [58]. In the present study, we observed an increase in presynaptic glutamate release within aIC, suggesting the possibility of the existence of insular pre-LTP during painful CP. Considering that both ACC and aIC are involved in emotional pain regulation, we propose that insular pre-LTP may also be involved in pain-related anxiety, which merits investigations in our future study.

### Neuromodulation techniques targeting IC as a viable therapy for painful CP

Generally speaking, glutamatergic pyramidal neurons and GABAergic interneurons are two major groups within IC [6]. Pyramidal neurons within superficial layers receive emotional nociceptive and visceral inputs from medial thalamus while those within deep layers project toward subcortical structures for descending pain control. Within local neurocircuitry, interneurons release GABA to inhibit insular output activity via feedforward inhibition [6, 12]. Jasmin L et al. observed that selectively activating insular pyramidal neurons within layer V via GABA_B_ receptor antagonist elicits hyperalgesia while inhibiting insular output activity via increasing local GABA content produces lasting analgesic effects in naïve rats [18]. In the present study, our functional evidence showed that TNBS treatment robustly elevated the activity of aIC pyramidal cells and chemogenetic inactivation of pyramidal neurons alleviated both hyperalgesia and reduced exploratory behavior in CP rats. Considering these, we concluded that aIC glutamateric neurons mediate hyperalgesia as well as pain-related anxiety under the condition of painful CP.

Another limitation of our study is that we did not examine whether activating insular neurons could elicit visceral pain in naïve rats owing to the limitation of our pain evaluating method. Since VFF probing is a measure of referred abdominal mechanical hypersensitivity when pancreatic inflammation invades the peritoneum [59], it may not be an efficacious index of internal visceral sensation under normal state. Thus, observing pancreas stimulation induced defensive behaviors via intra-abdominal electrodes [31] may be a better choice in our future studies to evaluate the sensory aspect of pancreatitis pain.

Historically, surgeries aiming at reducing ductal hypertension were introduced to treat painful CP in clinic. Nevertheless, it is not recommended in this day and age since ductal decompression failed to inhibit already existed central sensitization [60]. The cornerstone in the treatment of painful CP relies on multifunctional drugs, including opiates, antidepressants and antiepileptics, which provides favorable outcomes yet inevitably unbearable adverse effects, such as cognitive impairment and drug addiction [5]. Fortunately, the advent of brain stimulation, especially non-invasive paradigms including repetitive transcranial magnetic stimulation (rTMS) and transcranial direct current stimulation (tDCS), produces a promising alternative for modifying dysfunctional pain-matrix activity in the treatment of refractory pain disorders [61]. Recent technical improvements in the development of cooled double-cone coils in rTMS opened the possibility to stimulate deeper cortical regions, such as insula [62, 63]. Among various brain targets, such as prefrontal cortex and primary motor cortex [64, 65], IC is a newly-identified spot for the intervention of chronic pain. It is well-documented that insular stimulation relieves both experimentally-induced pain [62, 66, 67] and neuropathic pain [68]. The preponderant theory for this is high-frequency stimulation generated neural depolarization blockade [67, 69] or the recruitment of local GABAergic circuit [66], leading to functional inactivation of insular neurons. In the present study, we successfully relieved pancreatitis pain and pain-related anxiety in CP rats through chemogenetic inhibition of aIC neuronal activity. These data may lay preclinical basis for the application of insular stimulation in the remedy of pharmacoresistant pancreatitis pain, which may outperform motor cortex stimulation via targeting the affective component of pain.

In conclusion, the data provided in the present work pinpoint the role of cortical sensitization and modulation in pancreatic visceral pain, supporting insula, especially its anterior region (aIC), as a potential candidate alleviating visceral hyperalgesia and related affective disorders. Given the prosperity of the domain of neuromodulation this era has witnessed, insular stimulation may yield tremendous benefits for those suffering from visceral pain and should be considered by gastroenterologists in the treatment of CP.

## Data Availability

The datasets supporting the conclusion of this study are included in this article.

## Abbreviations

ACC: Anterior cingulate cortex
aIC: Anterior insular cortex
AMPAR: α-amino-3-hydroxy-5-methyl-4-isoxazole propionic acid receptor
AWT: Abdominal withdraw threshold
CP: Chronic pancreatitis
CREB: cAMP-response element-binding protein
fEPSP: Field excitatory postsynaptic potential
IBS: Irritable bowel syndrome
IC: Insular cortex
LTP: Long-term potentiation
NMDAR: *N*-methyl-D-aspartate receptor
pIC: Posterior insular cortex
PKA: Protein kinase A
POD: Post-operation day
PPF: Paired-pulse facilitation
sEPSC: Spontaneous EPSCs
TBS: Theta burst stimulation
TNBS: Trinitrobenzene sulfonic acid
VFF: *von* Frey filaments
